# Supplementary material for: Spatial-temporal differences and influencing factors of coupling coordination between urban quality and technology innovation in the Guangdong-Hong Kong-Macao Greater Bay Area
Source: PLoS One. 2023 Sep 21;18(9):e0289988. doi: 10.1371/journal.pone.0289988 (PMC10513345; doi:10.1371/journal.pone.0289988)
Supplement: Attachment — Submitted filename: Response to Reviewer1.docx [file pone.0289988.s001.docx]

Dear Reviewer,

Thank you very much for your time involved in reviewing the manuscript and your very encouraging comments on the merits. We have carefully revised the manuscript.

Comments: “Taking the Guangdong-Hong Kong-Macao Greater Bay Area as an example, this paper analyzes the temporal and spatial differences and influencing factors of the coupling and coordination of urban quality and technological innovation. The research topic has research significance. The research method of the article is scientific, and the analysis is detailed. These are worthy of recognition, but there are also some problems”

We also appreciate your clear and detailed feedback and hope that the explanation has fully addressed all of your concerns. In the remainder of this letter, we discuss each of your comments individually along with our corresponding responses.

To facilitate this discussion, we first retype your comments in italic font and then present our responses to the comments.

*Comment 1:*

*The biggest problem in this article is the lack of theoretical analysis. The article lacks a theoretical analysis mechanism for the relationship between urban quality and technological innovation. That is, before quantitative analysis, it is necessary to construct how the relationship between urban quality and technological innovation affects. The above leads to the following quantitative analysis. It is recommended to add this part.*

Response 1:

Thanks for your great suggestion on improving the accessibility of our manuscript. We have added the theoretical analysis mechanism in the third part of the paper. For your easy reference, we have added the following sections to the manuscript in red.

*Comment 2:*

*The analysis in the quantitative results part lacks theoretical support, and it is suggested to supplement it.*

Response 2:

Thank you for your suggestion, but I am not sure I understand your meaning correctly. We have reordered the quantitative results section and combined the comments of the experts for a more in-depth discussion, which have all been marked in red in the text. If there are still problems, please let us know, and we will be happy to make the article better!

*Comment 3:*

*The article did not carry out descriptive statistics on the variables used, including maximum value, minimum value, sample size, etc.*

Response3:

We strongly agree with you and have added this section to the text as follows:

**Table 3 .** Urban quality index descriptive statistics

| Variable | Obs | Mean | Std. Dev. | Min | Max |
| --- | --- | --- | --- | --- | --- |
| GDP per capita | 110 | 164220.49 | 344297.77 | 29683.882 | 3515051 |
| General public budget revenue | 110 | 20081.173 | 44529.541 | 499.778 | 199318.66 |
| Tertiary industry added value | 110 | .562 | .194 | .357 | 1.128 |
| Urbanization rate of the resident population | 110 | 34.573 | 27.849 | 1 | 84 |
| Total actual utilization of foreign capital per 10 billion yuan of GDP | 110 | 1.718 | 3.164 | .092 | 21.195 |
| Urban registered unemployment rate | 110 | 2.38 | .511 | 1.2 | 5.8 |
| Compulsory education teacher-student ratio | 110 | 2.38 | .511 | 1.2 | 5.8 |
| Public library holdings per capita | 110 | 1.094 | .716 | .055 | 2.792 |
| Energy consumption per unit of GDP | 110 | 1950.398 | 1615.899 | 97.804 | 6294.2 |
| Annual average concentration of fine particulate matter (PM2.5) | 110 | 36.128 | 12.131 | 15.7 | 66 |
| Environmental Protection Inputs | 110 | 32.175 | 53.254 | 0 | 331.635 |
| The number of public transport vehicles owned by 10,000 people in the city | 110 | 9.498 | 5.631 | 1.183 | 21.771 |
| The death rate of production safety accidents with a GDP of 100 million yuan | 110 | .064 | .05 | .009 | .22 |
| Economic externality | 110 | .207 | .166 | .005 | .596 |
| Number of hospital beds per 10,000 people | 110 | 33.627 | 10.145 | 15.146 | 56.783 |
| Number of doctors per 10,000 people | 110 | 22.772 | 5.778 | 13.065 | 46.002 |
| Per capita disposable income of urban residents | 110 | 100765.26 | 135387.17 | 19039.65 | 505875.63 |
| Increase in consumer price index | 110 | 1.392 | 1.413 | 0 | 6.11 |

**Table 4 .** Science and technology innovation descriptive statistics

| Variable | Obs | Mean | Std. Dev. | Min | Max |
| --- | --- | --- | --- | --- | --- |
| Internal expenditure on R&D expenses | 110 | 1966509.1 | 2783581 | 10596.662 | 15108100 |
| Number of R&D researchers | 110 | 53226.411 | 65145.153 | 711 | 345780 |
| Number of patents granted | 110 | 7498.073 | 12354.993 | 165 | 57050 |
| Number of publications | 110 | 54.7 | 31.621 | 1 | 109 |
| Financial Science and Technology Grant | 110 | 939.49 | 809.463 | 45.93 | 2988.86 |
| Information Infrastructure | 110 | 511381.25 | 987137.92 | 6000.161 | 5549818 |
| Number of students enrolled in general higher education institutions | 110 | 26106.064 | 36974.226 | 19 | 222412 |

*Comment 4:*

*This article lacks a discussion section, which explains the marginal contribution and research limitations of the article.*

Response 4:

Thanks for your great suggestion on improving the accessibility of our manuscript. We have added this part in the second part of the paper.And has been marked in red in the text, the main points are as follows:

The marginal contribution of this article is as follows: (1) This paper systematically analyzes the interaction mechanism between urban quality and science and technology innovation, summarizes the coupling and coordination effects of the two, and analyzes the evolution pattern of their interaction at the empirical level. (2) Since the statistical indicators of Hong Kong and Macao are not consistent with those of the mainland, there is no unified urban quality evaluation index system in the Guangdong-Hong Kong-Macao Greater Bay Area. Under the overall framework of the Guangdong-Hong Kong-Macao Greater Bay Area, this paper refers to the national standard "New Urbanization - Quality City Evaluation Index System" for the first time to portray the multi-attribute system characteristics of urban quality.

However, there are still some limitations to this study: At present, the article's research on the spatial structure and driving mechanism of the coupling coordination of urban quality and science and technology innovation is only limited to the Guangdong-Hong Kong-Macao Bay Area, and it can be extended to the national domain in the future to compare multiple city clusters.

*Comment 5:*

*The language section needs revision.*

Response 5:

Thank you for your comments. We have corrected the grammatical problems with the manuscript.

We would like to take this opportunity to thank you for all your time involved and this great opportunity for us to improve the manuscript. We hope you will find this revised version satisfactory.

Sincerely,

Yuxi Wu
