# Supplementary material for: Spatial-temporal differences and influencing factors of coupling coordination between urban quality and technology innovation in the Guangdong-Hong Kong-Macao Greater Bay Area
Source: PLoS One. 2023 Sep 21;18(9):e0289988. doi: 10.1371/journal.pone.0289988 (PMC10513345; doi:10.1371/journal.pone.0289988)
Supplement: Attachment — Submitted filename: Response to Reviewer5.docx [file pone.0289988.s002.docx]

Dear Reviewer,

Thank you very much for your time involved in reviewing the manuscript and your very encouraging comments on the merits. We have carefully revised the manuscript.

Comments: “Based on entropy TOPSIS, coupling coordination models, and other methods, this paper explored the spatial variation and influencing factors of the coupling coordination relationship between urban quality and technology innovation in the Guangdong-Hong Kong-Macao Greater Bay Area from 2011 to 2020. The article is relatively innovative in its research perspective, extensive in its content, and clear in its graphical presentation, but several important issues affect its publication”

We also appreciate your clear and detailed feedback and hope that the explanation has fully addressed all of your concerns. In the remainder of this letter, we discuss each of your comments individually along with our corresponding responses.

To facilitate this discussion, we first retype your comments in italic font and then present our responses to the comments.

*Comment 1:*

*The article has a large number of grammatical problems and unclear expressions, for example in the methodology section and the overview of the study area.*

Response 1:

Thank you for your criticism of the grammar of our article, we agree with you very much and we have re-examined the grammar of the article and revised it once again.

*Comment 2:*

*The article's theoretical value and practical significance are not significant, and the coupling relationship between urban quality and science and technology innovation is not very clearly expressed.*

Response 2:

Thanks to your suggestions, we have revised the mechanism of the coupling and coordination relationship between urban quality and technology innovation, and the revised content is as follows (and has been marked in the manuscript)：

Throughout the history of the world's urban development, the prosperity of cities cannot be separated from the progress of technology, and the generation of technology cannot be separated from the development of cities. The two systems of urban quality and technology innovation are independent and interconnected, mutually supportive and mutually constraining, maintaining structural stability and self-organizing ability. Coupling emphasizes that the two systems influence each other through internal or external effects, while coordination reflects how closely the two systems interact and adapt to each other. From the system perspective, the coupling and coordination of urban quality and science and technology innovation refer to the benign interaction between the two systems, resulting in a synergistic effect of 1+1>2, so that the overall function is greater than the sum of the functions of the two systems operating independently. Urban quality is an important condition in support of science and technology innovation, and science and technology innovation is the main driving force of urban quality. The two interact and promote each other, and when the development of one side is hindered, it will certainly affect the development of the other side.

On the one hand, city quality is the bearing system of regional space, which provides sufficient innovation factors and a superior innovation environment for science and technology innovation, effectively reduces the institutional threshold and communication cost of innovation subjects, and provides the necessary guarantee for the realization of science and technology innovation. The improvement of city quality means the improvement of residents' income level, the increase of employment opportunities, the optimization of the industrial structure an,d the improvement of environmental quality, which can expand the market demand for production and living services, further gather talents, capital and other innovation factors to operate and develop together in a certain area, attract different enterprises, universities and other innovation subjects to cooperate professionally and enhance the vitality of urban innovation. In addition, the optimization of city quality is inevitably accompanied by better infrastructure and information services, which can effectively reduce the communication barriers among innovation subjects, promote the exchange and transmission of knowledge, and also provide favorable conditions for the generation of regional innovation culture. At the same time, urban quality is an excellent practice place for new knowledge and technology, and the positive feedback of improved efficiency of urban space operation is a necessary basis for testing and improving innovation results, thus promoting the enhancement of science and technology innovation.

On the other hand, technology innovation is the output system of regional space, an important driving force for the coordinated development of economic, social, demographic, and environmental factors, and continues to optimize the spatial layout and operational efficiency of urban quality. Science and technology innovation undertakes the functions of cultivating innovative talents and researching technical knowledge, driving technology iteration and upgrading and industrial structure optimization in urban quality, and further restructuring of urban and rural areas, which can prompt the flow of innovation factors from labor-intensive industries to technology-intensive industries, thus improving labor productivity and investment yield of industries and optimizing the development mode of urban quality. At the same time, the application of innovations in healthcare and education will also promote more new industries to emerge, which promotes the quality of life of residents and the level of social services, and is conducive to the continuous improvement of urban quality.

*Comment 3:*

*Article title and main content need to be consistent. For instance， “technological innovation”、“Science and technology innovation”.*

Response 3:

Thank you for your suggestion, we are very sorry that we did not do a good job of unifying the subject words, we have checked the manuscript in detail and unified all the articles vocabularies.

*Comment 4:*

*The data sources are not clearly expressed in part, and some data need to be supplemented with specific websites and references. Also, how are the data for Macau and Hong Kong handled? There is the problem of exchange rate conversion..*

Response 4:

Thank you for your careful review of this article and your questions. The relevant amount of data involving the currency amounts of Hong Kong and Macau concerning currency representation in this article is converted according to the relevant rate of the current year's exchange rate. We have also added an elaboration in the article and added a data availability statement at the end of the article.

*Comment 5:*

*The indicator system of urban quality and technological innovation is not explained clearly. Some important references are missing. And the indicator system needs to be by the characteristics and actual situation of the Guangdong-Hong Kong-Macao Greater Bay Area.*

Response 5:

Similar to 4, we have added detail to the data issues in the text and added data availability statement at the end of the text. Thank you again for your suggestions.

*Comment 6&7:*

*The results analysis section is relatively weak, and no in-depth excavation has been carried out. In particular, there is no summary and practical analysis of the coupling characteristics and influencing factors of urban quality and technological innovation.*

*There are too many words in the discussion section, there are some contents that can be put inside the result analysis.*

Response 6&7:

Thank you for your suggestions. We have discussed your suggestions and modified the results and conclusion sections accordingly.

We would like to take this opportunity to thank you for all your time involved and this great opportunity for us to improve the manuscript. We hope you will find this revised version satisfactory.

Sincerely,

Fangfang Wang
