# Supplementary material for: Spatial-temporal differences and influencing factors of coupling coordination between urban quality and technology innovation in the Guangdong-Hong Kong-Macao Greater Bay Area
Source: PLoS One. 2023 Sep 21;18(9):e0289988. doi: 10.1371/journal.pone.0289988 (PMC10513345; doi:10.1371/journal.pone.0289988)
Supplement: Attachment — Submitted filename: Response to Reviewer8.docx [file pone.0289988.s003.docx]

Dear Reviewer,

Thank you very much for taking the time to review the manuscript and for your serious comments on our manuscript. We have revised the manuscript carefully.

To facilitate this discussion, we first retype your comments in italic font and then present our responses to the comments.

*(1)It is recommended to cite some references from 2023.*

Response 1:

Thank you for your suggestion about our references, we agree with you very much and have updated the references in the manuscript。

(2) *It is recommended to beautify Figure 1.*

Response 2:

Thanks to your suggestions, *We have updated Figure 1.*

*(3)The description of the study area should be as brief as possible.*

Response 3:

Thank you for your suggestion, and after discussion, we have changed the study area overview section of the manuscript, which now reads as follows:

The Guangdong-Hong Kong-Macao Greater Bay Area is located in the coastal region of South China (111°21′~114°53′E, 21°28′~24°29′N), and consists of the two special administrative regions of Hong Kong and Macau and the nine Pearl River Delta(PRD) cities of Guangzhou, Shenzhen, Zhuhai, Foshan, Zhongshan, Jiangmen, Huizhou, Dongguan, and Zhaoqing in Guangdong Province. The Guangdong-Hong Kong-Macao Greater Bay Area has a leading level of economic development, a complete industrial system, obvious cluster advantages, and the special advantages of "one country, two systems, and three customs zones," and has always been one of the regions with the highest degree of openness and economic vitality in China. In February 2019, the Central Committee of the Communist Party of China and the State Council issued "the outline of the development plan of the Guangdong-Hong Kong-Macao Greater Bay Area," making it clear that the Guangdong-Hong Kong-Macao Greater Bay Area will not only become a vibrant world-class city cluster, an international center of technology innovation, an important support for the construction of the Mainland, Hong Kong, and Macao in-depth cooperation demonstration area, but also a quality living circle that is pleasant to live, work, and travel, and become a model of high-quality development.

We would like to take this opportunity to thank you for all your time involved and this great opportunity for us to improve the manuscript. We hope you will find this revised version satisfactory.

Sincerely,

Fangfang Wang
